# Integral Projection Models and Sustainable Forest Management of *Agave inaequidens* in Western Mexico

**DOI:** 10.3389/fpls.2020.01224

**Published:** 2020-08-11

**Authors:** Ignacio Torres-García, Alejandro León-Jacinto, Ernesto Vega, Ana Isabel Moreno-Calles, Alejandro Casas

**Affiliations:** ^1^ Environmental Transdisciplinary Studies, Escuela Nacional de Estudios Superiores, Universidad Nacional Autónoma de México, Morelia, México; ^2^ Instituto de Investigaciones en Ecosistemas y Sustentabilidad, Universidad Nacional Autónoma de México, Morelia, México

**Keywords:** Agavoideae, mescal, demography, forest management, Michoacán, sustainability

## Abstract

In México, at least 37 *Agave* species are extracted from wild populations for producing distilled spirits. This activity involves harvesting mature agaves just before producing their inflorescences, which cancels sexual reproduction of plants used. The increasing demand of agaves spirits in national and international markets is determining a strong pressure on wild populations, most of them lacking adequate management. In addition, the dynamics of agave populations may be affected by natural phenomena like oscillation of rainfall regimes, which affects the recruitment of agave seedlings, or the scarcity of pollinators that may affect seed production and general population dynamics. We studied the demography of wild populations of *Agave inaequidens* to analyze critical conditions for populations recovery, modelling the effects of rainfall trends on the demographic performance of this species, and exploring response of populations to hypothetical extraction regimes and reforestation efforts. Our study was performed in four well-conserved wild populations in Central Western Mexico, each population was sampled in a plot of about one hectare composed by 10 subplots 50 x 5 m (2500 m^2^). Populations were monitored yearly between 2011 and 2013, measuring plant size, reproductive individuals, and fecundity. Data were analyzed through integral projection models by using the IPMpack for R, to perform prospective analyses. We in addition constructed stochastic models to explore the possible influence of rainfall variation on species demography, using data for the drier and wetter years of the study period. Population growth varied from λ=1.003 to λ=0.899 among populations and years, and exceptionally λ=0.559 after a fire event. Low rainfall decreases λ values, indicating especial limitations to harvesting agaves during dry years whose frequency most probably will increase. In general, extraction rates from 10% to 30% of mature individuals are viable to maintain λ above 1, and these rates may be higher if new plants are introduced in populations. Depending on levels of extraction, our models suggest that it is necessary to carry out actions of reforestation, and *in situ* management according to the trends found in each site. This is one indispensable condition to maintain λ close to or greater than 1. Sustainable extraction of wild agaves is possible, some communities are already carrying out a repertoire of goods practices in this direction, but together with ecological criteria and good management techniques, strict regulations and social organization are needed to achieve it.

## Introduction

Mexico is the territory that hosts the higher species diversity of the genus *Agave* in the world (García-Mendoza et al., 2019). It is the main setting of natural evolutionary divergence of this genus and the principal area of management and domestication of agaves (Colunga-GarcíaMarín et al., 2017). The interactions between such diversity and the extraordinarily rich human cultures inhabiting this territory started thousands of years ago. Archaeobotanical remains, dating back nearly 10,000 years B.P., witness these long interactions (McNeish, 1967; Gentry, 1982), including gathering, incipient management of populations and, eventually, domestication of some species. The most ancient uses recorded like food, rope making and textile manufacturing, are all current. A total of 22 use categories of agaves (Torres-García et al., 2019) are practiced in rural Mexican societies. In the past two centuries some of these uses were developed in immense industries, such as the case of the sap-fermented beverage called pulque, henequen fibre, tequila and more recently the mescal, raicilla, and other agave spirits industries. Some of these activities have experimented a boom and then a collapse (Bowen and Valenzuela-Zapata, 2009; Colunga-GarcíaMarín et al., 2007; Ramírez-Rodríguez, 2018; Valenzuela-Zapata and Macias-Macias, 2014). In the last three decades, diverse negative socio-environmental phenomena related to the boom and intensification of production, trade, exportation, and consumption of mescal are evident. The Mexican regulatory organisms and official norms that rule the tequila and mescal appellations of origin (DOT and DOM for their acronyms in Spanish, respectively), have allowed a vertiginous incursion of investors into the traditional communities that produce mescal, which has determined substantial changes in their forms of production and marketing of this beverage. These changes have determined a strong pressure over rural mescal producers that dramatically increases the extraction of agaves from forests and harvesting agave crops intensively cultivated. Among the main negative trends it is now possible to see the depletion of wild populations and natural habitats, the transformation of both forests and traditional crop fields to establishing agave monocultures maintained with chemical inputs, which, in the case of *Agave tequilana* var. *azul* F.A.C. Weber, are called “blue deserts” (Torres-García et al., 2019). These phenomena are generated by the demand of mescal in the big cities of Mexico and the international markets. Another negative consequence of the intensive extractive regimes is the alteration of interactions in biotic communities, for instance, the removal or damage to nurse plants, and impact on flower visitors. Thus, the foraging patterns of the main pollinators of agaves, bats, and diverse communities of birds and insects that visit agave flowers and have a role in pollination, are all being affected by over-extraction of mature agaves, which compromises the pollination effectivity and the general biological diversity in numerous ecosystems where the agaves occur (Rocha et al., 2006; Trejo-Salazar et al., 2016). It has been documented by Estrella-Ruiz (2008) and Baraza-Ruiz and Estrella-Ruiz (2008) that in areas where the offer of nectar decreases, bats scarcely visit them, and move to populations with a higher nectar offer. This fact determines that populations offering few flowers are scarcely visited and seed production is low or unlikely.

In México, 37 *Agave* species have been documented to be extracted exclusively from wild populations for producing distilled spirits, mainly adult plants just before sexual reproduction, most of them without targeted management (Torres et al., 2015a). The traditional process of elaborating mescal involves four stages, the first one is the harvest, a critical step determining the amount and quality of mescal and the recovering of populations used for production. The second stage is baking agave stems, in which carbohydrates of the plant tissue are lysed into smaller molecules and the smoked flavour is acquired. The third stage is fermentation, in which sugar is transformed into alcohol. And finally, distillation, through which the alcohol of mescal is separated. Depending on the species used, the weight and concentration of carbohydrates may vary, as well as the efficiency of the distillation methods and the preferences of tastes that are related to alcohol concentration. And all these aspects influence the variable amount of mescal that can be obtained per individual plant of agave. In our fieldwork, and based on information from mescal producers, we have recorded that variation may range from 0.5 litres per agave in *A potatorum* Zucc., *A. marmorata* Roezl., and *A. karwinskii* Zucc., 2.0 litres in *A*. *maximiliana* Baker, 2.0 to 15.0 litres in *A. rhodacantha* Trel., 4.5 litres in *A*. *cupreata* Trel. & A. Berger, and 10 litres in *A*. *angustifolia* Haw.

In Western Mexico, at least nine *Agave* species have been used to produce distilled beverages which, depending on the area they occur and are used, receive different local names like “raicilla”, “tepe”, “tutsi”, “barranca”, “tusca”, “vino de cerro”, “vino de mezcal”, among others, all of them grouped in the generic term mescal. The main species involved in these distillates are *A. tequilana* var. *azul*, *A. rhodacantha*, *A. americana* var. *subtilis* (Trel.) Valenz.-Zap. & Nabhan that are domesticated and cultivated, as well as *A. angustifolia*, *A. maximiliana*, *A. durangensis* Gentry, *A. bovicornuta* Gentry, *A. cupreata*, and *A. inaequidens* K. Koch., which grow wild in regional forests.

We studied the species *A. inaequidens*, which belongs to the Crenatae group of the Agavoideae (Gentry, 1982; APG III, 2009), a group of species well known for their rare or null production of clonal shoots, being the sexual monocarpic event their only way of reproduction (Gentry, 1982). Throughout their life cycle, individuals of these agaves accumulate carbohydrates, especially in their stems and foliar bases, a process that prepare the plants to sexual reproduction and end their life, using most of their reserves to generate massive floral panicles, flowers, nectar, pollen and, after pollinated, seeds. Agaves of this species reach maturity in 12 to 25 years depending on the substrate and exposure to solar radiation where plants grow (Torres-García, 2016). On average, a mature individual of *A. inaequidens* may produce nearly 400,000 fertile seeds. However, to produce mescal this unique event is cancelled by cutting the floral shoot just before it begins to develop. We have estimated that only in five municipalities, of northern Michoacán, nearly 12,000 *A.* *inaequidens* individual plants are harvested from wild populations every year (Torres et al., 2015b), which cancels the production of approximately 4.8 billion seeds. This activity, therefore, severely affects the regional populations and their potential to regenerate after harvesting such high number of reproductive organisms.

In this study, we focused our attention on analyzing the population dynamics of *Agave inaequidens* in order to establish ecological criteria for recommending sustainable forms of using mature plants for mescal production. Our study, therefore, looks for understanding population biology of this and other agave species, as well as supporting the construction of regulations and designing strategies and environmental education programmes for using and conserving agaves of this species and others with similar life history traits and problems. Not only human impact on reproduction may affect the dynamics of agave populations. For instance, previous studies estimated that oscillation of rainfall regimes affects the recruitment of agave seedlings (Jordan and Nobel, 1979; Nobel, 1992). These studies documented that episodic events of high precipitation, which also determined episodes of high recruitment. In addition, the scarcity of pollinators associated to both natural and human factors may affect seed production and the general population dynamics (Torres et al., 2015a). In some species, amounts of humidity and presence or not of nurse plants may determine the success or not of seedlings recruitment and the maintenance of populations regeneration (Rangel-Landa et al., 2015). Therefore, documenting the biological processes determining reproduction and the indispensable conditions for ensuring seedling establishment, survival and growth are relevant for identifying management patterns needed for conservation of agaves (Torres et al., 2013; Torres et al., 2015a).

Demographic studies of non-timber forest products (NTFP) have been useful for identifying crucial aspects of management based on ecological criteria for sustainable use. A high number of studies on ecological bases for sustainable management of NTFP have found in demography a useful tool not only for analyzing theoretical problems in relation to adaptability, vulnerability and conservation ecology, but also as a way to analyze consequences of impacts of human activities on populations and ecosystems and, importantly, a helpful approach to design strategies for sustainable use of biotic resources (Shackleton et al., 2015). Studies on monocarpic perennial plants have been carried out for several species (Metcalf et al., 2003; Metcalf et al., 2006), which emphasize that the reproductive strategy of these plants is directly related to the individuals size, larger plants yielding larger numbers of seeds and likewise increasing the number of seedlings. Case studies using matrix models of *Agave* species under human exploitation are relatively scarce [e.g., *Agave cupreata* (Illsley et al., 2007), *A. marmorata* (Jiménez-Valdés et al., 2010), *A. potatorum* (Torres et al., 2015a), and *A. angustifolia* (Arias-Medellín et al., 2016)], compared with the large number of species used. However, the few studies available for this genus allow identifying some general demographic patterns. The most remarkable aspects are that survival and growth are the processes that mostly contribute to the finite growth rate (λ), whereas fecundity has low contribution. Studies available also coincide that management actions are urgent on populations that are over-exploited, in order to procure maintaining the demographic equilibrium and contribute to the conservation of these valuable resources. Management actions such as identifying harvest thresholds of reproductive individuals based on ecological criteria, as well as protection of individuals of the lower size categories, and enhancing recruitment of other stages have been proposed for some species (Martin et al., 2011; Torres et al., 2015a). In addition, some studies emphasize the need of protecting particular nurse plants, which are crucial for ensuring establishment of agave species depending on interactions of facilitation (Torres et al., 2013; Rangel-Landa et al., 2015). However, matrix models study the populations performance commonly by using discrete categories based on the researcher methodology and may not represent satisfactorily the biological cycle of a species.

The studies referred to above have mainly used matrix models (Caswell, 2001; Ramula et al., 2009; Gaoue and Ticktin, 2010; Zuidema et al., 2010; Schmidt et al., 2011; Gaoue et al., 2011). But more recently, integral projection models (IPM´s) that do not categorize populations into discrete stages but use data on continual actual plant sizes have been used to study the demography of some NTFP species (Ellner and Rees, 2006; Merow et al., 2014). However, hitherto, these models have not been used for analyzing population dynamics of *Agave* species (Kuss et al., 2008). In this study we use these models for analyzing populations of *A. inaequidens* and the conditions for sustainable use of its populations. We studied the demography of wild populations of *A. inaequidens* to analyze critical conditions for populations recovery, the effects of rainfall trends on the demographic performance and responses of populations to hypothetical extraction regimes and reforestation efforts. In addition to population biology topics, this study aspires to generate information useful to agave managers and decision makers.

## Methods

### Study Species

*Agave inaequidens*, locally named “maguey bruto” or “maguey alto”, is a rosetophyllous monocarpic species that forms medium to large-sized rosettes. It is the most abundant agave species in the Trans-Mexican Volcanic Belt, inhabiting areas at elevation ranges from 1850 to 2600 m (Torres-García, 2016). Its main reproductive system is sexual, but it may have axillary asexual propagules, whose reproduction may be active in response to damages of the apical meristem, but this type of events is rare. Its flowers are mainly pollinated by bats (*Leptonycteris* spp.), orioles (*Icterus* spp.) and some insects (*Apis mellifera*) (León, 2013). Studies on reproduction of other species of the Crenatae group suggest that this species most probably is self-incompatible. One single capsule produces on average 205 ± 75 (n=57) viable seeds, a panicle is composed on average by 21 ± 10 (n=16) umbels, and reproductive individuals produce on average 417,356 ± 202,176 (n=27) viable seeds (León, 2013). According to mescal producers who sow agave seeds and take care of their plantations, depending on the site where plants are established *A. inaequidens* may reach maturity in 12 to 25 years. In addition to humidity and soil quality, solar radiation received by a plant may influence its size and reproductive capacity (Nobel and Valenzuela, 1987; Nobel, 2003). The studied species grows mainly in temperate habitats such as subtropical scrub, oak forest, pine-oak forest and fir forest, the populations mainly having an aggregated spatial distribution in patches called “magueyeras”, but in some areas it may show scattered spatial distribution (Gentry, 1982; Torres-García, 2016). This agave represents a multipurpose resource for numerous rural communities. Torres et al. (2015b) and Valenzuela-Zapata et al. (2011) documented that this agave is used for about 34 different specific uses, among them the extraction of complete individuals for producing the distilled spirit mescal, the most important cultural and economic activity currently carried out with this species.

**Figure 1:**
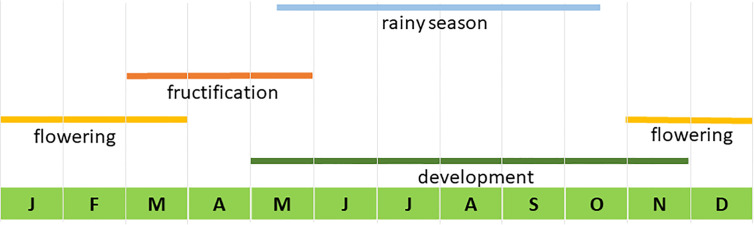
Temporal phenology of Agave inaequidens in the study area. Modified from León, 2013.

Traditional production of mescal with *A. inaequidens* in the study area is carried out from October to May, the dry season. Rain limits agave harvest and mescal production because it hinders the movement of donkeys that carry the harvested agaves, wets the wood used to bake agaves, and floods the rustic roasting pits. According to León (2013), the reproductive phenology of *A. inaequidens* (**Figure 1**) starts with the emergence and development of inflorescences, starting in May and ending in November. When the inflorescence is emerging and it is no more than 2 m tall, the managers cut the inflorescence to stop its development, a practice called “capado” or castration. But the stems are not harvested until October. People harvest reproductive individuals and those that they identify that will bloom the following year, to complete a batch. One batch is composed on average by 147 agaves (n=29 producers) (Torres et al., 2015b). To complete a batch, people harvest agaves from different sites, including plantations, backyard fences, home gardens, managed and unmanaged forests, within their community or bought agave stems to other communities. The proportion of agaves from these sites is highly variable, depending on their availability and social arrangements. To produce one litre of mescal from this species, 25 to 30 kg of agave are needed. A harvested individual (stem and foliar bases), or “piña”, weights on average 70 to 80 kg, meaning that about two litres of mescal can be obtained from one individual agave.

### Study Sites

This study was carried out in Michoacán (**Figure 1**), in Central Western Mexico, where we selected four well-conserved wild populations, without signs of extraction. Once we obtained the permit by local authorities, we established permanent study plots of nearly 1 ha each. Each plot was divided into 10 parallel subplots of 50 x 5 m (2,500 m^2^), each subplot was marked with metal labels and geo-positioned to make easier further visits. Each study plot was monitored for two consecutive years or periods. Ahead, we show the results of monitoring per period (first or 1 monitored period and second or 2 monitored period).

### Permanent Plots

**Figure 2:**
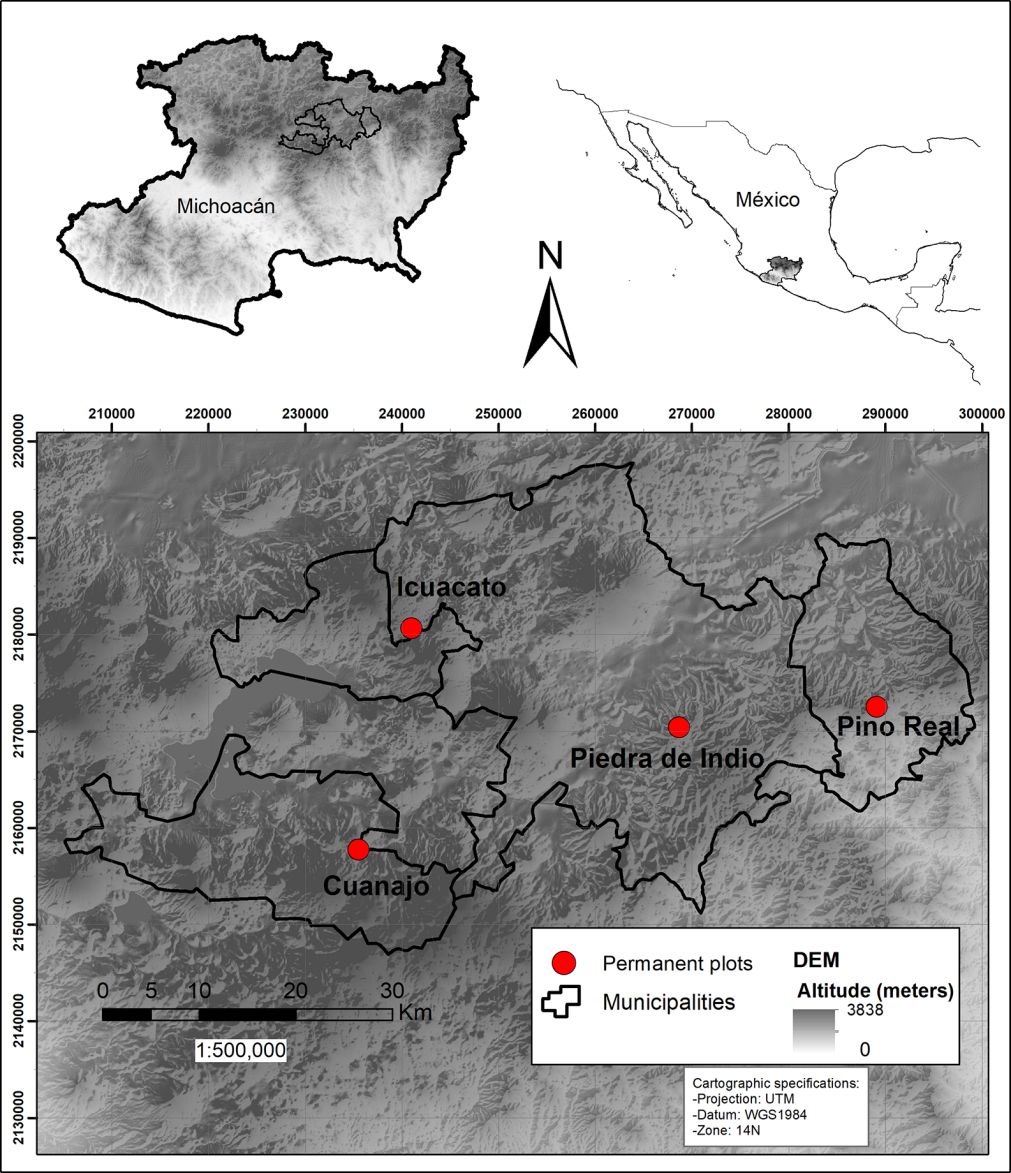
Map of the study area and the location of the four permanent plots in the Trans-Mexican Volcanic Belt.

1. Piedra de Indio is a natural protected area of the municipality of Morelia, located at elevations around 2414 m, with a northern exposure, the slope inclination varying from 30° to 35° (UTM 14Q 268684-2170422), where vegetation is *Pinus-Quercus* forest. The plot studied in this site will be called ahead “Piedra de Indio”. 2. The Ejido Pino Real in the municipality of Charo, where vegetation is also *Pinus*-*Quercus* forest, at elevations around 2349 m, with north-northeastern exposure, the slope inclination varying between 15° and 30° (UTM 14Q 289118-2172533). This plot will be called “Pino Real” throughout the text. 3. The Ejido Cuanajo in the municipality of Pátzcuaro, where vegetation types are *Quercus* and *Pinus*-*Quercus* forests, at elevations around 2541 m, with south-southeastern exposure, and slope inclinations varying between 35° and 45° (UTM 14Q 235534-2157777). This plot will be further called “Cuanajo”. 4. The Ejido of Quiroga in the municipality of Quiroga, where vegetation is *Quercus* forest, at elevations around 2475 m, with south-southeastern exposure, the slope inclination varying from 15° to 25° (UTM 14Q 235534-2157777). This plot will be called “Icuacato” (**Figure 2**). The annual mean precipitation in the two first plots is 1360 mm and in the other two is 1200 mm. We obtained this information from WorldClim BIO12 (Fick and Hijmans, 2017).

### Data Collection and Characterization of Size

The four populations were monitored yearly, between 2011 and 2013. We used the method of Total Foliar Area (TFA) developed by Torres et al. (2015a) for characterizing plant size in demographic studies of *Agave potatorum*. This method consists in direct measurement of the rosette size, which is used to characterize the functional state of the individuals. The TFA is calculated by measuring length and width of four leaves of four different whorl levels, then estimating the average leaf area based on calculating an ellipsoid area, the geometrical form of leaves. The average area of the ellipsoid (AAE) was then multiplied by the leaves number (LN). Therefore, the TFA is the product of AAE * LN. To adjust the data to a normal distribution, these were transformed into their natural logarithm (logTFA), similarly as in other demographic studies of monocarpic perennials (Metcalf et al., 2003). Therefore, in the three monitoring times of our study, the following data were recorded for each agave plant: Number of leaves, length, and width of four leaves of different strata of the rosette. The individuals and leaves measured were labelled and consecutively numbered.

### Reproduction and Fecundity

Based on studies on reproductive phenology by León (2013), we identified the seasons of development, flowering, and fructification of the panicles (**Figure 1**), and we designed a strategy to characterize the seeds production. The first step was to estimate the number of capsules per umbel. A total of 120 umbels from all studied populations was counted, and we recorded on average 96 fruits per umbel. Then, for estimating the average number of seeds per capsule, we sampled 57 capsules from three of the populations studied (“Cuanajo”, “Icuacato” and “Piedra de Indio”). After capsules dehiscence, we counted the total number of seeds per fruit, identifying viable and unviable seeds by their color. Based on previous experiences we knew that black seeds were viable whereas those with clear color were not. We calculated on average a 375 ± 73 seeds per capsule, and about 36,000 seeds per umbel. In each plot, during the monitoring events, we recorded the number of reproductive individuals, the number of umbels per panicle and the estimated number of seeds per flowering individual.

The second step to estimate fecundity, was carrying out experiments of natural establishment in each population, determining the proportion of seeds that were established one year after their germination. The experiments were established *in situ*, using two gridded (grid square size: 50 cm) subplots (5 x 2.5 m) beneath reproductive individuals, which are areas influenced by seed rain. The number of seeds, viable and unviable were counted in each subplot. One month later, the seedlings with cotyledon inside the subplots were recorded in 2012. One year later, the number of one leaf seedlings established in 2013 was recorded. Unfortunately, in three of the populations these experiments were altered by livestock and confident results were obtained only from one population. The successful subplots experiments were those established in the population “Piedra de Indio”. This rate was included in the IPM´s of the four populations since it was the only real-confident data that we could obtain.

### IPM´s

Critical life history traits of *Agave inaequidens* were characterized to construct the IPM´s. This plant species is monocarpic, reproducing only by sexual means. During their development, plants grow continually increasing their volume, accumulating carbohydrates. When mature, these reserves are directed to produce a massive inflorescence, flowers, nectar, and seeds. The size of the panicle is proportional to the plant size. *A. inaequidens* is pollinated mainly by *Leptonycteris* bat species (León, 2013). The reproduction causes the death of an agave, but also the production of hundreds of thousands of seeds, and the recruitment rate will depend on the sites and substrate where seeds arrive, the amount of seed predation, and the intensity of precipitation. For this agave, at least two months of high humidity favor germination of practically all viable seeds that were not predated; therefore, the formation of seed banks is unlikely, similarly as in almost all studies in this topic conducted with *Agave*.

After conducting surveys of the annual population dynamics, IPM´s were constructed using the IPMpack package for R (Easterling et al., 2000; Ellner and Rees, 2006; Metcalf et al., 2013; Ortiz-Rodríguez, 2015; R Development Core Team, 2010). This analysis is based on a kernel representing the growth probabilities between continuous size stages that are conditioned by the survival and offspring production. In this study, populations are structured by a set of continuous variability, according to the TFA values of individual plants. For analyzing such structure, we used the following equation:

(1) $$\begin{array}{c} n(y,t+1)=\int_{L}^{U}K(y,x)n(x,t)dx\\ =\int_{L}^{U}[P(x,y)+F(x,y)]n(x,t)dx \end{array}$$

Where *n*(*y,t* + 1) is the size distribution of the established and recruited plants at time *t* + 1; *n*(*x,t*) is the size distribution of plants at time *t*; *L* and *U* are the IPM in the lowest and highest size limits. The kernel *K* is divided into the two sub-kernels *P* and *F*. The *P* sub-kernel representing the growth and survival transitions, the *F* sub-kernel describing the *per-capita* contribution of the reproductive individuals given the recruitment density in the next census. To build *K*, the growth, survival, and fecundity functions of *P* and *F* were calculated from regression models based on the data recorded for each population and period (see **Table 1** and **Appendix 1**). The model was then built by using the midpoint rule (Ellner and Rees, 2006; Zuidema et al., 2010) in order to achieve a numerical integration and obtaining a matrix of 100 x 100 dimensions.

**Table 1:** Models of the demographic processes used to construct IPM´s for the first (1^st^) and second (2^nd^) monitoring periods (first and second consecutive years of the study, respectively).

Site (monitoring period)	Survival	Growth	Fecundity	Density/size range
**Cuanajo (1^st^ period)**	y~x+x2: D^2^ = 0.051; AIC=267.94	y~x+x2+x3: D^2^ = 0.94; AIC=165.48	y~x: D^2^ = 0.441; AIC=40.176	480 ind TFA (cm^2^) 3–126,950 (log) TFA 0.52–5.10
**(2^nd^ period)**	y~x+x2: D^2^ = 0.056; AIC=248.69	y=0.08708x^2^+0.36079x R^2^ = 0.85	Null	
**Icuacato** **(1^st^ period)**	y~x+x2: D^2^ = 0.154; AIC=339.59	y=0.93871x R^2^ = 0.85	y~x: D^2^ = 0.44; AIC=40.716	524 ind TFA (cm^2^) 1–154,608 (log) TFA -0.29–5.19
**(2^nd^ period)**	y~x+x2+x3:D^2^ = 0.117; AIC=469.37	y~x+x2+x3: D^2^ = 0.83; AIC=491.91	y~x+x2: D^2^ = 0.41; AIC=62.597	
**Piedra de Indio** **(1^st^ period)**	y~x+x2: D^2^ = 0.176; AIC=119.37	y=0.94086x R^2^ = 0.959	y~x: D^2^ = 0.288; AIC=25.129	158 ind TFA (cm^2^) 1–190,336 (log) TFA 0.80–5.28
**(2^nd^ period)**	y~x: D^2^ = 0.161; AIC=87.277	y=0.70846 x^2^+0.03731 x R^2^ = 0.967	Null	
**Pino Real** **(1^st^ period)**	y~x+x2: D^2^ = 0.158; AIC=57.287	y~x+x2+x3: D^2^ = 0.937; AIC=-30.812	y~x: D^2^ = 0.367; AIC=11.488	137 ind TFA (cm^2^) 10–15,225 (log) TFA 1–5.18
**(2^nd^ period)**	y~x: D^2^ = 0.007; AIC=39.412	y=0.96131 x R^2^ = 0.979	y~x+x2: D^2^ = 0.99; AIC=6.0126	

Densities correspond to the number of individuals in 2,500 m^2^ sampled units, and the range size of individuals were calculated based on Total Foliar Area (TFA) and logarithm TFA. x= plant size at time t.

### Prospective Analysis

#### Elasticity Analyses

Through the models referred to above, we performed elasticity analysis (De Kroon et al., 1986; Metcalf et al., 2013) to identify which plant sizes have a stronger effect on the λ values per site and year.

#### Numerical Simulations of Management Strategies

Based on information about traditional forms of management of *A. inaequidens* (Torres et al., 2015b), we carried out numerical simulations of their possible effects on population dynamics. We simulated the effect of *in situ* management actions *sensu* Blancas et al. (2010), such as tolerance of reproductive individuals, their extraction at different rates, and the enhancement of young plants (individuals 6 to 12 months old grown in nurseries), under different introduction efforts. Simulations were conducted combining variables and scenarios to characterize the effect of different management strategies on λ. Firstly, we calculated the basic *K* of each population, and then simulations were performed in two nested cycles. The external cycle simulates the extraction of 0 to 100% reproductive plants through 5% intervals. The internal cycle simulates different introduction efforts from 25 to 300 young plants through intervals of 25 plants. The external cycle affects the *K* diagonal of the reproductive plants, which represents the survival. The value of the diagonal was reduced in 5% intervals, then the percentage of reduction was calculated and subtracted from the initial value. The internal cycle affects the population vector, which allows identifying the sizes of young individuals that should be reintroduced; the total number of plants to be introduced was divided by the number of categories. To calculate the number of plants per category, the augmented categories were added to the initial population vector. With this modified vector and *K*, we calculated λ for the resulting populations by using 100 iterations.

#### Influence of Rainfall Variation

To explore in a simple way the possible influence of rainfall variation on species demography, data from the nearest climatic stations to each population were analyzed. To find out if a rainfall series could be represented with a pdf Gamma, we firstly fitted the series to a Gamma distribution (fitdistr function in MASS package) and then, through a test of Kolmogorov-Smirnoff, we determined if the series correspond to the pdf gamma (ks.test function in stats package). When data did not adjust to a Gamma probability, we used a pdf normal (see **Appendix 2**).

For representing two possible, simple, scenarios of change of rainfall patterns (one drier than the other), we used rainfall data recorded during the period when the demographic data were collected. We used the rainfall thresholds corresponding to the first and third years of sampling (519.8 mm and 744.7 mm, respectively). We calculated simulations of 1,000 events of rainfall for each population studied and for each precipitation threshold using parameters of the fitted pdf. For each condition we identified the number of events below (nb) and over (na) the threshold.

We performed projections of each population using the series of simulated rainfall. The demographic model used was: *n*(*t*) = *Mx* * *n*(*t* – 1) where n is the vector of the population sizes. The kernel *Mx* used depends on the value of the simulated rainfall. If the rainfall value at the time (t) is lower than the threshold, we used the kernel of the dry period, but if it is higher, we used the kernel of the wet period. The stochastic growth rate was calculated as: $\lambda rand=avg(\frac{n(t)}{n(t-1)},$ considering the last 500 iterations of the series of populational vectors n(t).

For characterizing the effect of the variation of the rainfall simulations over the management strategies, we used the numbers of events below (nb) and over (na) the threshold and the kernels simulating the extraction and introduction in the analyzed populations.

The average matrix was calculated as: *Mavg* = ((*ml* * *na*) *+* (*m*2 * nb))*/*(*na+nb*) where m1 and m2 are the kernels of management for each annual period analyzed. With the average matrix it was possible to calculate the variation $\left\{d1=na*{\left(ml-Mavg\right)}^{2};d2=nb*{\left(m2-Mavg\right)}^{2};v=\frac{di+d2}{na+nb}\right\}$ and the standard error $sd=sqrt(v);se=\frac{sd}{sprt(na+nb)}$ of each kernel for each population and rainfall threshold.

#### Integrated Stochastic Rainfall Model

To provide broader scenarios of environmental patterns and to provide general helpful recommendations to agave managers, with the two rainfall patterns studied we calculated two average kernels with the four individual kernels for each period. We looked for obtaining a general view of how management strategies modify growth rates.

## Results

### Population Dynamics: IPM´s

In the population Cuanajo, a total of 480 agave plants were monitored. In the first period 16 agaves bloomed and the analysis projected λ=0.975 for the period 2011-2012, and λ= 0.899 for the period 2012-2013, when no plants bloomed. In the population Icuacato, a total of 524 agaves were monitored. In the first period six mature agaves bloomed and λ= 1.003 was projected; 10 agaves bloomed in the second one; λ=0.559 as consequence of wildfire that severely affected plants of the smaller sizes.

In the population Piedra de Indio, 158 agave plants were monitored, three of them bloomed and λ=0.976 was projected, but no plant flowered in the second period and had λ=0.955. In the population Pino Real a total of 137 agaves were monitored. In the first period one agave bloomed and the population had λ= 0.952, and in the second period one agave bloomed and the population had λ= 0.966.

### Prospective Analysis

#### Elasticity Analysis

**Figure 3:**
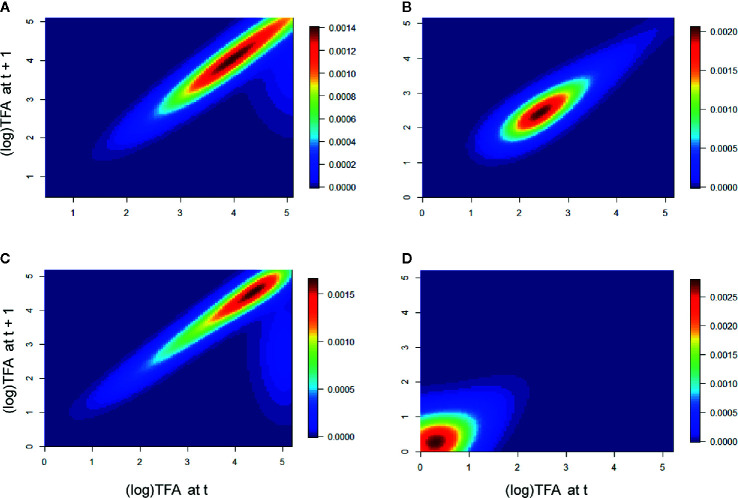
Elasticity of the populations Cuanajo and Icuacato. Cuanajo **(A)** first period, **(B)** second period. Icuacato **(C)** first period, **(D)** second period (wildfire). Warmer colors represent size stages that mostly contributed to λ.

In the population Cuanajo, during the first period of the analysis we found that the individuals of intermediate and larger sizes had a greater effect on λ, whereas the intermediate stages were more relevant in the second period. In the population Icuacato, the larger stages were more relevant in the first period and the smaller stages during the second period that suffered the wildfire (**Figure 3**).

**Figure 4:**
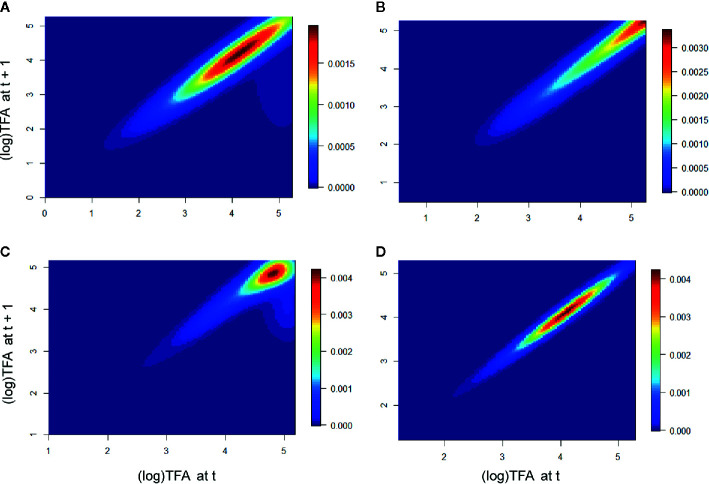
Elasticity of the populations Piedra de Indio and Pino Real. Piedra de Indio **(A)** first period, **(B)** second period. Pino Real **(C)** first period, **(D)** second period. Warmer colors represent size stages that mostly contributed to λ.

In the population Piedra de Indio, the individuals of intermediate and larger stages had a greater effect on λ in the first period, whereas the larger stages were more relevant in the second period. In the population Pino Real, the larger stages were more important in the first period, whereas the intermediate and the larger intermediate stages were relevant in the second period (**Figure 4**).

#### Management and Population Dynamics Simulations

**Figure 5:**
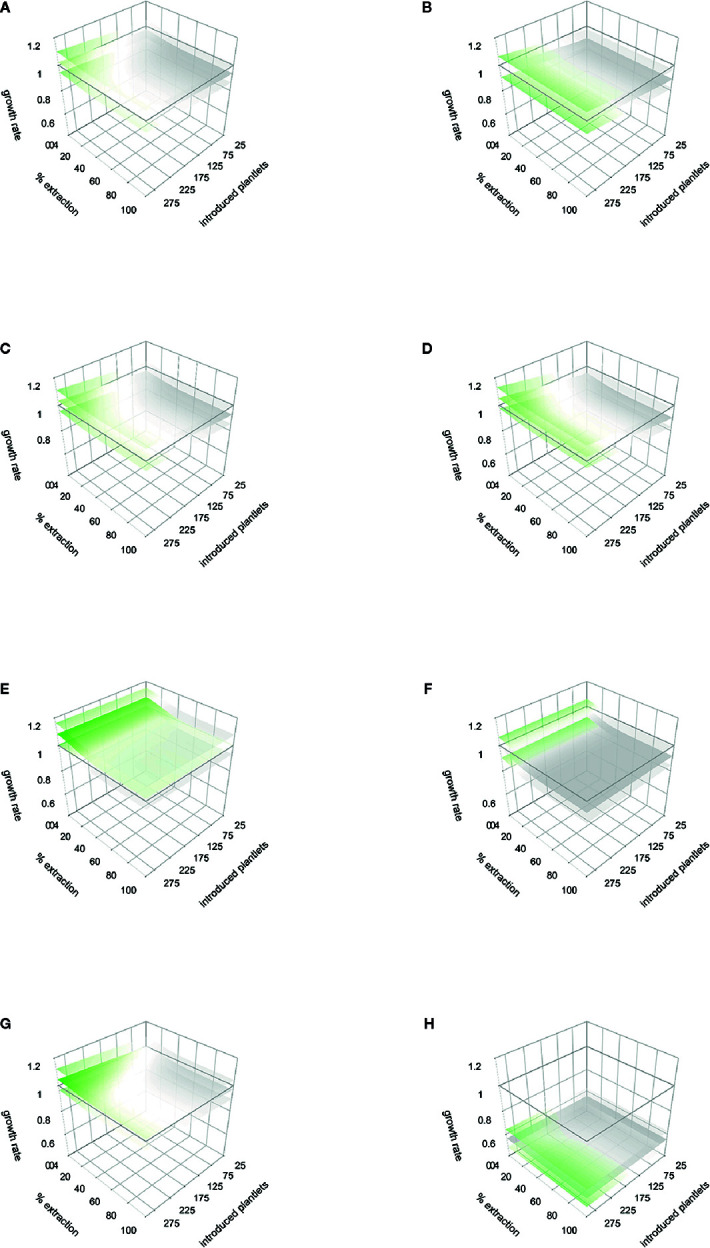
Response to management simulations and stochastic rainfall models for the two monitored periods (first and second years of the study, respectively). Cuanajo **(A)** first period, **(B)** second period. Icuacato **(C)** first period, **(D)** second period (Forest fire). Piedra de Indio: **(E)** first period, **(F)** second period. Pino Real: **(G)** first period, **(H)** second period. Values of λ below 1 are gray colored, values equals 1 are white colored, and λ values over 1 are green colored. Lower and upper λ responses to the lower and upper rainfall thresholds are represented in shaded sheets.

For the population Cuanajo, during the first period studied, the projected management simulations showed that extraction rates from 10% to 30% of mature individuals may maintain λ > 1, only if 200 to 300 agave plants of the lower sizes (plantlets 6 to 12 months old grown in nurseries), are introduced into the population, respectively (**Figure 5A**) (**Table 2** shows the amount of mature agaves, that represent the percentage of extraction). For the second period, no combination of introduction and extraction allowed to maintain λ > 1 (**Figure 5B**).

**Table 2:** Different combinations of thresholds of management actions to maintain λ above unity in the population Cuanajo during the first monitoring period of the study.

Management action	First period		
**Extraction of mature individuals**	10% 26 ind.	20% 52 ind.	30% 78 ind.
**Reforestation (number of plantlets needed)**	200	250	300

In the population Piedra de Indio, during the first period, the extraction rate of 5% of mature individual would maintain λ > 1 if more than 200 agaves of the lower sizes are reintroduced to the population, and up to 60% if > 300 agaves are introduced into the population (**Figure 5C** and **Table 3** show the amount of mature agaves representing the percentage of extraction). In the second period, the extraction of 5% would be sustainable if more than 200 young agaves are introduced to the population, and from 40% up to 100% if > 275 young agaves are introduced into the population (**Figure 5D**, and **Table 4** show the amount of mature agaves, that represent the percentage of extraction).

**Table 3:** Different combinations of thresholds of management actions to maintain λ above unity in the population Piedra de Indio during the first monitoring period of the study.

Management action	First period				
**Extraction of mature individuals**	5% 2 ind.	15% 8 ind.	25% 14 ind.	30% 16 ind.	60% 33 ind.
**Reforestation (number of plantlets)**	200	225	250	275	300

**Table 4:** Different combinations of thresholds of management actions to maintain λ above unity in the population Piedra de Indio during the second monitoring period of the study.

Management action	Second period		
**Extraction of mature individuals**	20% 11 ind.	35% 19 ind.	40%–100% 22–56 ind.
**Reforestation (number of plantlets needed)**	225	250	275

For the population Pino Real, the simulations showed that for the first period that an extraction rate of 20% to 40% of mature individuals would maintain λ > 1 if more than 25 to 275 agaves are introduced in the population (**Figure 5E** and **Table 5** show the amount of mature agaves that represent the percentage of extraction). In the second period no combination of management strategies allowed to maintain λ > 1 (**Figure 5F**).

**Table 5:** Different combinations of thresholds of management actions to maintain λ above unity in the population Pino Real during the first monitoring period of the study.

Management action	First period
**Extraction of mature individuals**	20%–40% 11–22 ind.
**Reforestation (number of plantlets)**	25 to 275

For the population Icuacato, in the first period studied, an extraction rate of 5% of adult plants can be compensated if more than 50 young agaves (6 to 12 months old) are reintroduced into the population, an extraction rate of 10% of adult plants may be sustainable if more than 75 young plants are reintroduced, and up to 100% extraction of adult plants if 275 or more young agaves are introduced to the population; all these scenarios would maintain λ > 1 (**Figure 5G**, and **Table 6** show the amount of mature agaves that represent the percentage of extraction). For the second period, no combination of introduction and extraction allowed to maintain λ > 1 (**Figure 5H**).

**Table 6:** Different combinations of thresholds of management actions to maintain λ above 1 in the population Icuacato in the first monitoring period.

Management action	First period						
**Extraction of mature individuals**	10% 9 ind.	20% 18 ind.	25% 22 ind.	30% 27 ind.	40% 36 ind.	50% 45 ind.	100% 91 ind.
**Reforestation (number of plantlets needed)**	75	100	150	175	200	250	275

#### Stochastic Rainfall Model

The projected variation of the numerical simulations in the stochastic rainfall models renders or reproduces the average patterns (i.e., the magnitude of variation does not change depending on the extent of exploitation or reintroduction). Upper rainfall thresholds projected a higher λ value, except in population Pino Real (**Figure 5** upper shaded sheets, **Table 7**). These models showed that management simulations may be influenced by rainfall patterns, in some cases lower rain scenarios determine that no extraction would be recommendable. In the first period (**Figure 5A**), we can find combinations of extraction and reintroduction that produce growth rates greater than one. In general, in all the populations, to maintain λ in equilibrium or above one, is needed a combination of low extraction with reintroduction of many individuals. However, in the population of Pino Real (scenario A), it seems that the population could support a maximum extraction rate of 20% to 40%, regardless of introduction rates. The numerical simulations on scenario B suggest that growth rates are lower than one.

**Table 7:** Growth rate values (λ) (average, variation, and standard errors) in populations at different rainfall thresholds.

Site	Rainfall threshold (mm of annual rain)	Avg growth rate (λ)	SE	λ >1	λ <1
**Cuanajo**	519.8	1.1376	0.0121	599	401
**Cuanajo**	744.7	1.2090	0.0145	602	398
**Icuacato**	519.8	1.1230	0.0103	624	376
**Icuacato**	744.7	1.2142	0.0149	497	503
**Piedra**	519.8	1.0472	0.0057	562	438
**Piedra**	744.7	1.0848	0.0090	557	443
**Pino Real**	519.8	0.9916	0.0013	7	993
**Pino Real**	744.7	0.9677	0.0025	76	924

The last two columns show the number of times the λ value showed in the model. The sum of the two values equals 1,000, the simulation size.

#### Integrated Stochastic Rainfall Model

**Figure 6:**
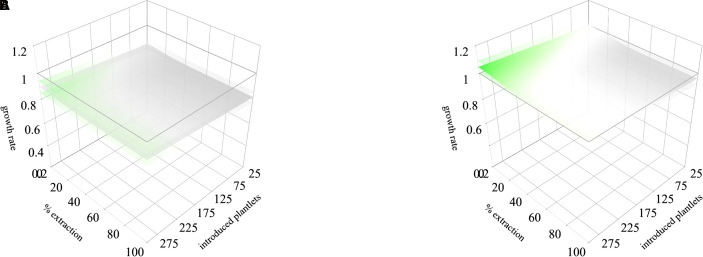
Integrated stochastic rainfall models for the two monitored periods (first and second years of the study, respectively. The scenario **(A)** corresponds to the first monitored period and scenario **(B)** corresponds to the second monitored period. Lower and upper lambda responses to the lower and upper rainfall thresholds are represented in shaded sheets. Lower λ values are in gray, and higher in green.

A general scenario can be projected from the average models for general recommendations. In the first period, a threshold of 10% to 30% of extraction combined with an introduction of 175 to 300 individuals can be performed to maintain λ above 1 (**Figure 6A**). This model showed also that management simulations may be influenced by rainfall patterns, in some cases, scenarios with lower rainfall determine that no extraction would be recommendable. In the second period, no combination of management actions allows to maintain λ above 1 (**Figure 6B**). This pattern may be influenced by the integration of the kernel of the second period of Icuacato population were the wildfire occurred.

## Discussion

### Population Dynamics and Elasticity

Our models showed that in the first period of the study, λ is close to the population equilibrium, i.e., the mortality rate is balanced with the recruitment rate and if those conditions are maintained, the populations would remain stable throughout time. The elasticity analyses showed that for all models we constructed, the alterations in the vital rates of the larger size categories, i.e., the nearly mature and already mature agaves that are harvested for mescal production have greater effects on λ, as it is shown in our prospective analyses. Only in the population Pino Real, λ values, in both study periods, had values slightly below the equilibrium, suggesting a decreasing trend, but these values were not far from the unity. Similarly as found in other works with *Agave* species of semiarid zones of Mexico (Illsley et al., 2007; Jiménez-Valdés et al., 2010; Torres et al., 2015a; Arias-Medellín et al., 2016), survival and growth are the vital rates that mostly contribute to λ in *A. inaequidens*, but in this study we found that fecundity also showed a significant contribution to λ, a fact that can be explained based on findings by Metcalf et al. (2003). These authors documented demographic patterns of monocarpic perennials and analyzed them as a function of individual’s size, finding a direct relationship between plant size and the number of seeds and seedlings established. *A. inaequidens* has larger individuals than other species whose demography has been studied (*A. angustifolia*, *A. cupreata*, *A. marmorata*, and *A. potatorum*), and in addition, it occurs in temperate zones with higher rainfall regimes than the semiarid areas where the other species are distributed. The greater the number of seeds in a more favorable environment, and the higher fertility values are expected to contribute to the population dynamics of this species.

*A. inaequidens* is totally dependent on its reproduction *via* seeds, unlike other agave species that have active asexual propagation and produce stoloniferous shoots during their growth, as in the cases of *A. tequilana*, *A. angustifolia*, and *A. salmiana*. This trait has defined that the management and propagation of those species, predominantly through the transplantation of suckers, which has determined unnecessary to allow individuals blooming to obtain seeds. This management type allows to obtain agaves with the same characteristics of the mother plant, a useful aspect when seeking to unify production. However, it has great repercussion on decreasing genetic variation, which in turn determines low capacity of resistance against pests and effects of climate change. Exclusive sexual reproduction of *A. inaequidens* allows and enhances the conservation of high levels of genetic variation, as shown in recent studies (Figueredo et al., 2015; Figueredo-Urbina et al., 2017).

The stochastic rainfall models as well as the integrated rainfall model help to identify how much oscillations in rainfall may affect the growth of agaves populations. As general trends, these models suggest that dry years would be particularly severe for harvesting reproductive agaves. The unpredictability of rainfall can be used as a justification for lowering harvest rates, in order to maintain agave populations in the long term. But in addition, this research line deserves special attention since climate change may be increasing the variation of climate regimes (IPPC, 2015).

### Implications for Sustainable Extraction

Regional *Agave inaequidens* managers and mescal producers in northern Michoacán, practice several management strategies, which represent a gradient of management complexity that goes from simple gathering to intensive *ex situ* cultivation. *In situ* management in wild populations in forests is carried out by a small number of managers. In those populations, people use to transplanting juvenile agave plants from undesirable to desirable places, weeding competing plants, let standing mature individuals for seed production and natural dispersal. They, in addition, collect seeds and sow them in rustic nurseries, and some of the resulting plantlets are targeted to *ex situ* cultivation, but some others are destined to *in situ* reforestation efforts (Torres et al., 2015b). Our findings of the previous and current studies suggest that, considering the number of agaves per batch of mescal production, (on average 147 mature agaves), and based on our prospective models, each population have different extraction thresholds. The main recommendation, based on our models and the integration of the rainfall patterns in wild populations, is to harvest in a precautionary threshold of 10 to 30% of mature individuals in areas of 2,500m^2^, tolerating individuals, preferably the larger ones, since seeds production is larger in these individuals, to maintain natural seed production and recruitment of new individuals. We recommend emphatically to let standing larger individuals, particularly of this species, since an historical decrease in the size of wild individuals have been documented as a result of harvesting the larger individuals for mescal production (Torres et al., 2015b). In this way, promoting that larger individuals’ reincorporation to the populations, would determine higher yield of seeds and stems to the mescal production. This threshold must be accompanied with the sowing of seeds, nursing, and introduction agave plantlets to wild populations from 175 to 300 individuals. These reforestation efforts must be carried out just before the rainy season starts in order to enhance the survival of this younger agaves.

Communities of managers of *Agave inaequidens*, and producers of mescal in northern Michoacán are rural “ejidatarios”, and their territories comprise hundreds of hectares with wild populations of this species, which means that a greater area than at present could be managed, and the impact on populations would be buffered if more populations are used extracting fewer individuals per population. Since managers relay only on wild populations to complete a batch (the rest are agaves from plantations and other agroforestry systems), the harvesting thresholds discussed could be used as a guide in different populations within the communitarian territories. Other aspects are relevant when discussing social and economic aspects of the problems related to using agaves. For instance, the diversification of species used, the diversification of sources of agaves, the diversification of activities supporting local households’ economy, and, importantly, diversifying markets looking for fair principles of commercialization, are all important issues for designing sustainable alternatives.

We have identified the average number of mature agaves to complete a batch, and the variation of environmental conditions (interannual and long-term variations) and their effects on populations dynamics. In addition, we have identified market pressures motivating increasing extraction of mature agaves and responses of management techniques developed by people to attenuate the effects of extraction on forests. Cultivation of agaves emerged as an alternative, but cultivators of agave plantations have drastic economic problems, because of plant mortality, pests, and slow growth of agaves. These factors have led people to use chemical inputs, which has determined economic and ecological negative impacts. We are therefore before a complex socio-ecological system that requires social and ecological holistic approaches to be faced. We have documented details of the management techniques developed by local people, and these can be consulted in our study (Torres et al., 2015b). All these techniques are valuable tools for solving problems, and our ecological studies, some of them reported here, provide elements to complement such techniques, considering biological and ecological factors that may help to finer adjustments of the techniques for specific situations. But particularly important are the social agreements and regulations for an organized way of using common resources. Basic principles proposed by Ostrom (1990); Dietz et al. (2003); Ostrom et al. (2012), as well as the valuable experiences of social organization proliferating in Mexican rural communities and ejidos are of great value. This is for instance the case of the organization *Sansekan Tinemi* and those of other organizations of Nahua people from Guerrero (Martin et al., 2011; Illsley et al., 2018). Therefore, academic ecological studies find in local people’s organizational experiences the possibility to produce knowledge useful to answer scientific questions but also to solve problems.

## Conclusions

Depending on the different levels of extraction and the areas needed to supply of raw material for the production of distilled spirits, our models suggest that it is necessary to carry out actions of reforestation, and *in situ* management of agaves according to the trends found in each site. This is one indispensable condition to maintain λ close to or greater than 1. The models also indicate that management actions must be specific, according to characteristics of each population, like density and structure, and the fluctuating climatic conditions.

The accelerated demand of mescal production and the patterns of exploitation that have predominated, determine in some cases a drastic depletion of wild agave populations. In some of the analyzed localities, these conditions drive to carry immediate management actions. Although our models were carried out in particular systems and climatic conditions for the sampling years, and has limitations in characterizing fecundity, there is an urgent need to implement comprehensive management actions. It is feasible to propose actions and monitoring their success or the responses of populations to such management practices. And, according to the patterns found in these monitoring activities, to continue or to reconsider adjustments of actions, based on an adaptive management framework principles (Christensen et al., 1996).

## Data Availability Statement

All the datasets generated for this study can be found by asking to the first author.

## Author Contributions

IT-G: Conception and design of the research, fieldwork gathering data, data analysis, writing the manuscript, and reviewing several versions of manuscript. AL-J: Fieldwork gathering data. EV: Analysis design, data analyzing, analysis interpretation, and writing the manuscript. AM-C: Writing the manuscript and reviewing of manuscript. AC: Conception and design of the research, analysis interpretation, and writing and reviewing several versions of manuscript.

## Funding

We thank the Dirección General de Asuntos del Personal Académico (DGAPA) for the postdoctoral scholarship awarded to the first author. Also, for financial support to the PAPIIT projects IN206217 and IN206520.

We also thank the Consejo Nacional de Ciencia y Tecnología CONACYT for financial support to the project A1-S-14306, and to the Comisión Nacional para el Conocimiento y Uso de la Biodiversidad (CONABIO) for supporting the project RG023.

We also thank the Red Temática de Sistemas Agroforestales de México (RedSAM) project 293348 and the Red Temática de Productos Forestales No Maderables (RED-PFNM), project 293914 of CONACYT for the support provided to this research.

## Conflict of Interest

The authors declare that the research was conducted in the absence of any commercial or financial relationships that could be construed as a potential conflict of interest.

The reviewer [AM-L] declared a shared affiliation, though no other collaboration, with the authors [IT-G, AL-J, EV, AM-C, AC] to the handling Editor.
