# Supplementary material for: Integral Projection Models and Sustainable Forest Management of Agave inaequidens in Western Mexico
Source: Front Plant Sci. 2020 Aug 11;11:1224. doi: 10.3389/fpls.2020.01224 (PMC7438764; doi:10.3389/fpls.2020.01224)

Appendix 1. Statistical models based on the data recorded for each population and period. Population Cuanajo first period (a, b,c), second period (d, e, f)

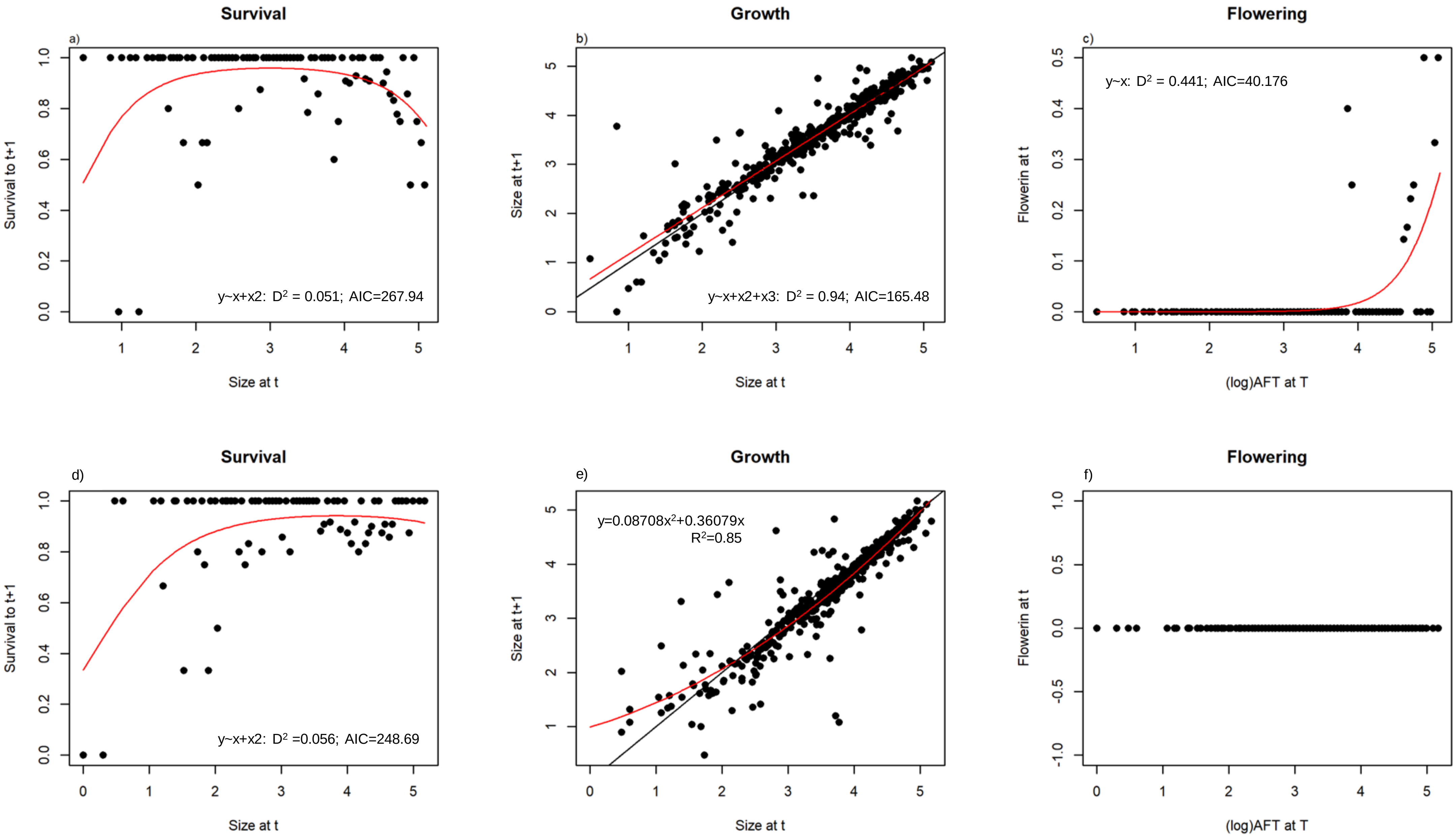

Population Icuacato first period (a, b,c), second period (d, e, f)

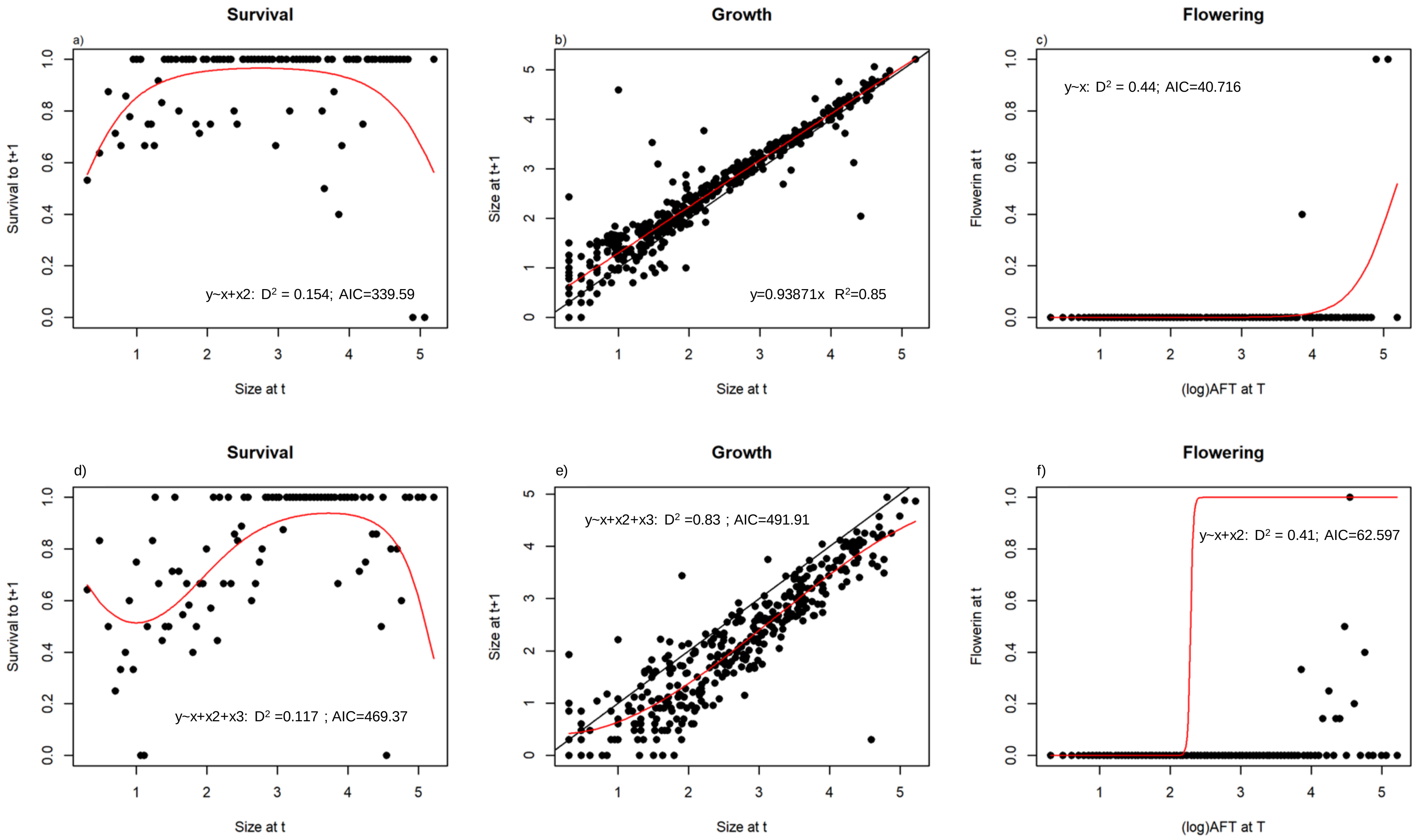

Population Piedra de Indio first period (a, b,c), second period (d, e, f)

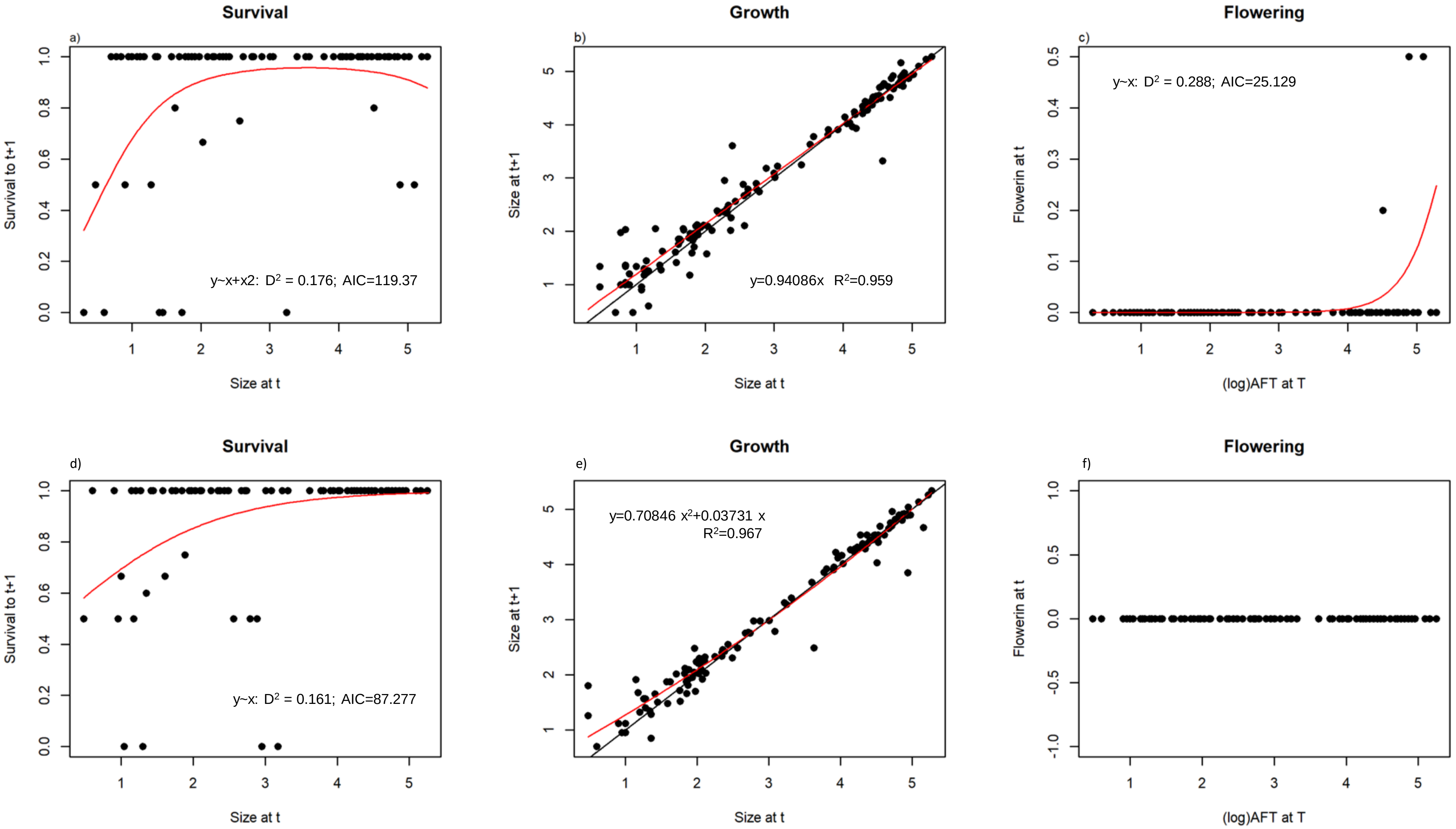

Population Pino Real first period (a, b,c), second period (d, e, f)

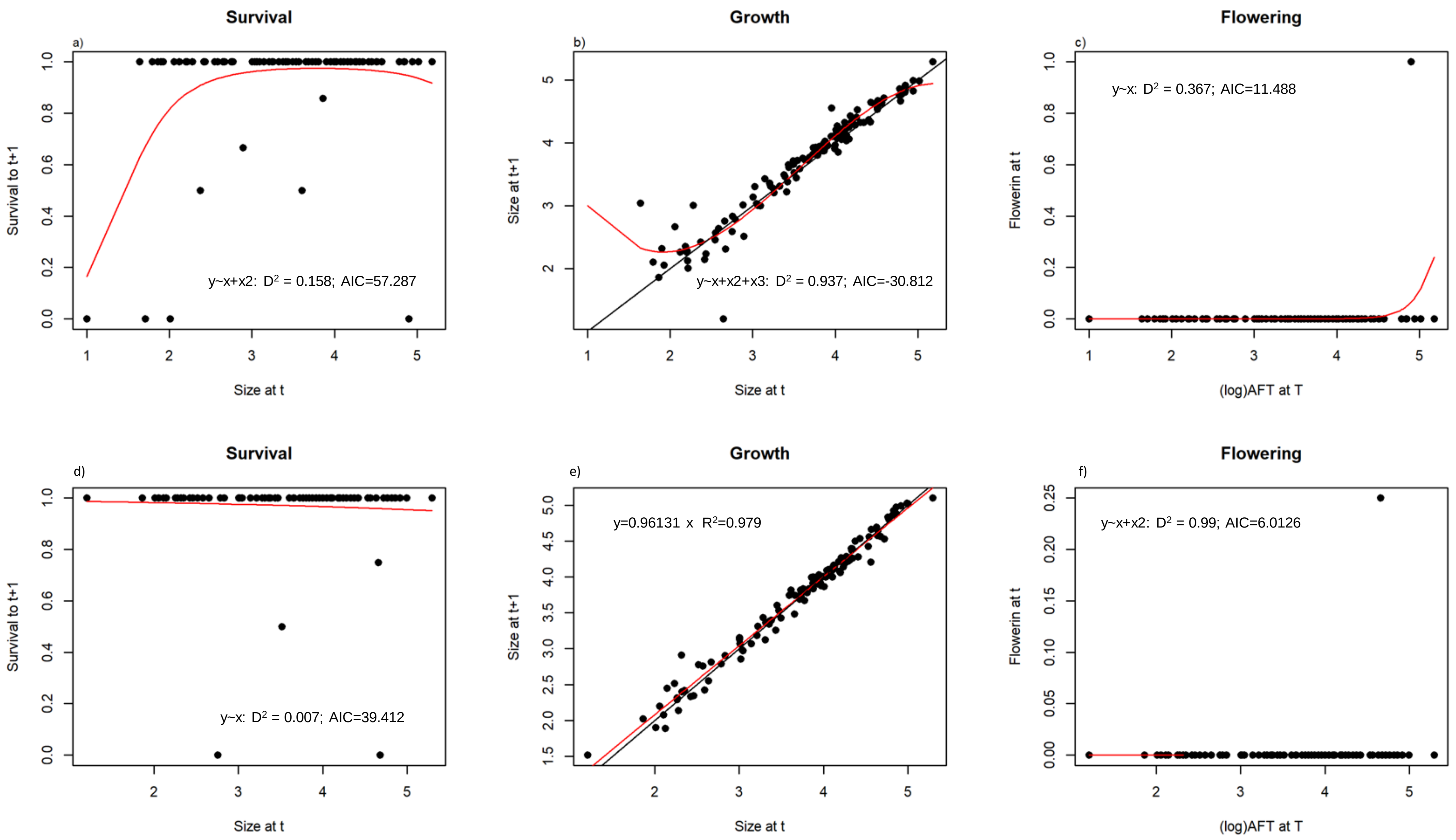

Supplement: Supplementary file 1 [file DataSheet_1.pdf]
