# Supplementary material for: Integral Projection Models and Sustainable Forest Management of Agave inaequidens in Western Mexico
Source: Front Plant Sci. 2020 Aug 11;11:1224. doi: 10.3389/fpls.2020.01224 (PMC7438764; doi:10.3389/fpls.2020.01224)
Supplement: Supplementary file 2 [file DataSheet_2.pdf]

Appendix 2.

Kolmogorov-Smirnoff test for probability density function (pdf) selection in four locations.
Null hypothesis states that data can be modelled with the pdf tested.
P values <0.05 rejects the H0.

Klomogorov-Smirnoff Test for a Gamma pdf

Location	shape	rate	D	pval
Cuanajo	2.4810	0.0029	0.2890	**0.0011**
Piedra	1.9632	0.0021	0.2008	**0.0389**
Pino	8.6375	0.0064	0.1594	0.1343
Icuacato	15.7712	0.0226	0.1063	0.7712

Klomogorov-Smirnoff Test for a Gaussian pdf

Location	mean	sd	D	pval
Cuanajo	863.0465	369.5821	0.1547	0.2298
Piedra	923.1404	428.1407	0.0938	0.7672
Pino	1348.8731	376.4369	0.0937	0.7264
Icuacato	698.4167	180.0367	0.1277	0.5567
