# Supplementary figures and images for: Integral Projection Models and Sustainable Forest Management of Agave inaequidens in Western Mexico
Source: Front Plant Sci. 2020 Aug 11;11:1224. doi: 10.3389/fpls.2020.01224 (PMC7438764; doi:10.3389/fpls.2020.01224)

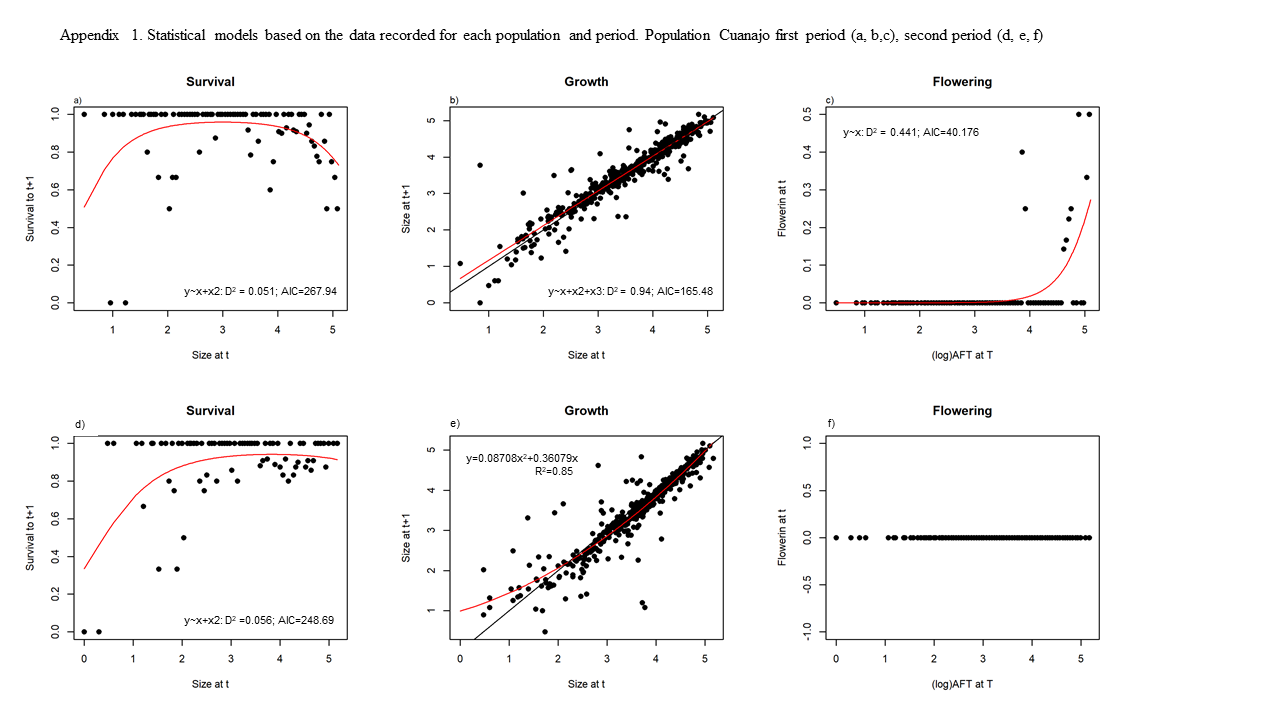

Supplement: Supplementary file 3 [file Image_1.tif]

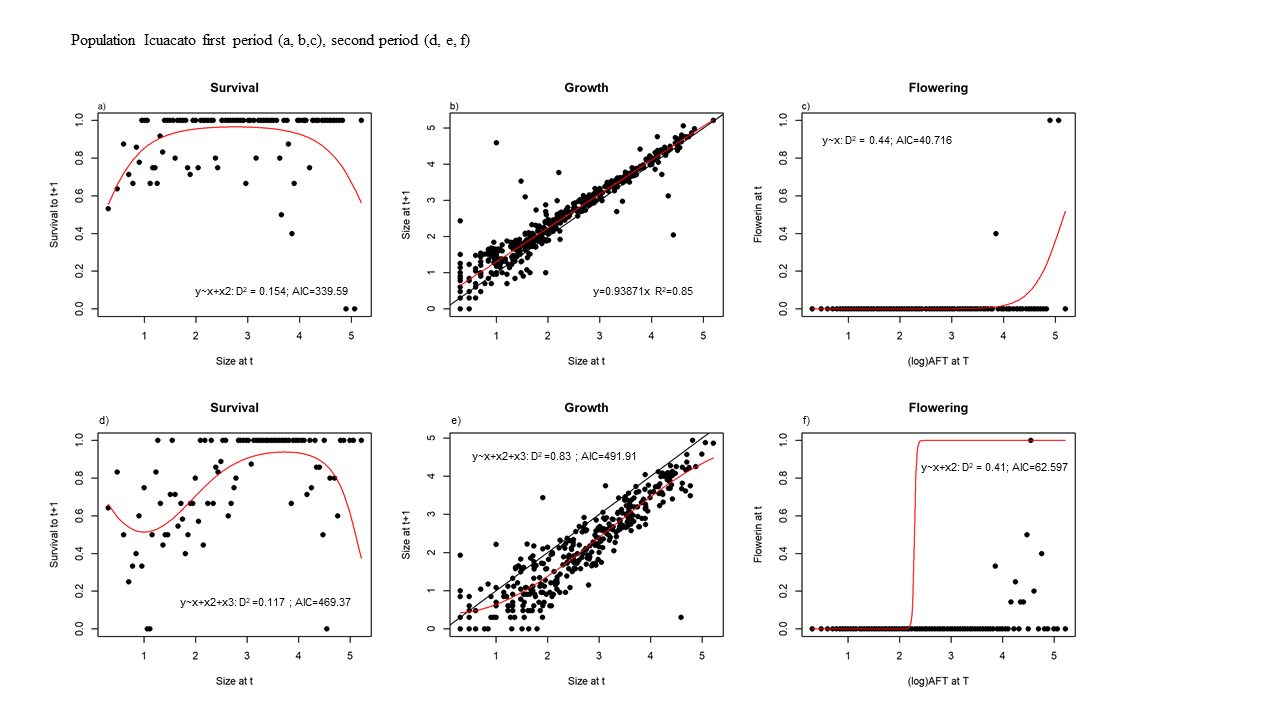

Supplement: Supplementary file 4 [file Image_2.tif]

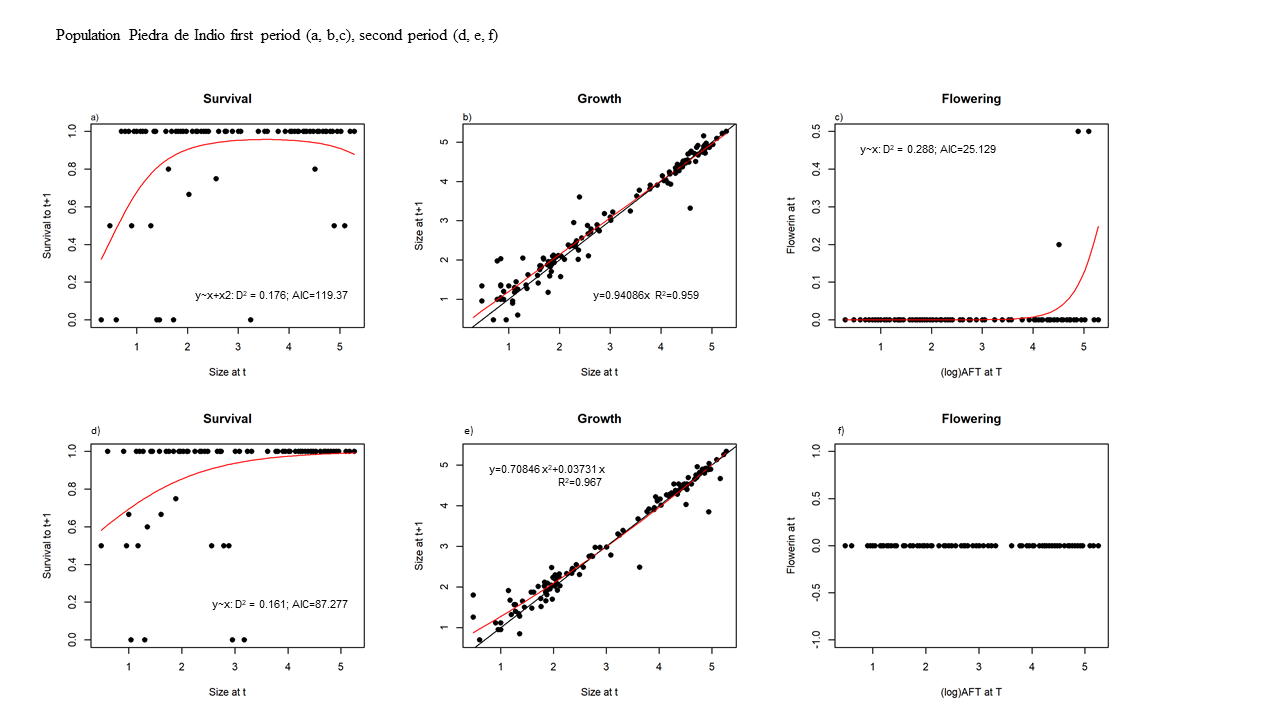

Supplement: Supplementary file 5 [file Image_3.tif]

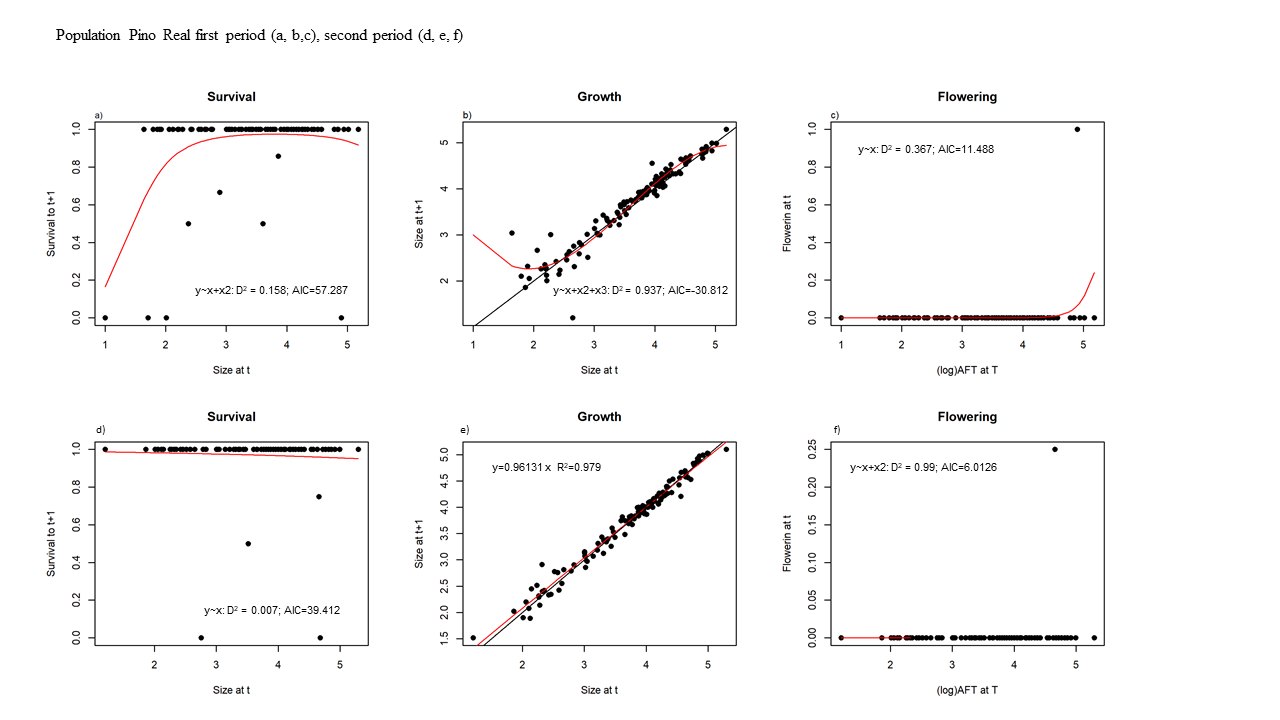

Supplement: Supplementary file 6 [file Image_4.tif]
